# A Serious Puzzle Game to Enhance Adherence to Antirheumatic Drugs in Patients With Rheumatoid Arthritis: Systematic Development Using Intervention Mapping

**DOI:** 10.2196/31570

**Published:** 2022-02-18

**Authors:** Bart PH Pouls, Charlotte L Bekker, Sandra van Dulmen, Johanna E Vriezekolk, Bart JF van den Bemt

**Affiliations:** 1 Department of Rheumatology Research Sint Maartenskliniek Nijmegen Netherlands; 2 Department of Pharmacy Radboud Institute for Health Sciences Radboud University Medical Centre Nijmegen Netherlands; 3 Department of Primary and Community Care Radboud Institute for Health Sciences Radboud University Medical Centre Nijmegen Netherlands; 4 Netherlands Institute for Health Services Research Utrecht Netherlands

**Keywords:** medication adherence, serious game, eHealth, rheumatoid arthritis, intervention mapping, intervention development

## Abstract

**Background:**

Patients’ implicit attitudes toward medication need and concerns may influence their adherence. Targeting these implicit attitudes by combining game-entertainment with medication-related triggers might improve medication adherence in patients with rheumatoid arthritis (RA).

**Objective:**

The aim of this study was to describe the systematic development of a serious game to enhance adherence to antirheumatic drugs by using intervention mapping.

**Methods:**

A serious game was developed using the intervention mapping framework guided by a multidisciplinary expert group, which proceeded along 6 steps: (1) exploring the problem by assessing the relationship between medication adherence and implicit attitudes, (2) defining change objectives, (3) selecting evidence-based behavior change techniques that focused on adjusting implicit attitudes, (4) designing the intervention, (5) guaranteeing implementation by focusing on intrinsic motivation, and (6) planning a scientific evaluation.

**Results:**

Based on the problem assessment and guided by the Dual-Attitude Model, implicit negative and illness-related attitudes of patients with RA were defined as the main target for the intervention. Consequently, the change objective was “after the intervention, participants have a more positive attitude toward antirheumatic drugs.” Attention bias modification, evaluative conditioning, and goal priming were the techniques chosen to implicitly target medication needs. These techniques were redesigned into medication-related triggers and built in the serious puzzle game. Thirty-seven patients with RA tested the game at several stages. Intrinsic motivation was led by the self-determination theory and addressed the 3 needs, that is, competence, autonomy, and relatedness. The intervention will be evaluated in a randomized clinical trial that assesses the effect of playing the serious game on antirheumatic drug adherence.

**Conclusions:**

We systematically developed a serious game app to enhance adherence to antirheumatic drugs among patients with RA by using the intervention mapping framework. This paper could serve as a guideline for other health care providers when developing similar interventions.

## Introduction

Rheumatoid arthritis (RA) is an autoimmune disease characterized by symmetric chronic polyarthritis which, if untreated, leads to pain, joint damage, and decreased quality of life [1,2]. The cornerstone of RA treatment is the use of antirheumatic drugs (disease-modifying antirheumatic drugs [DMARDs]), which reduce disease activity, radiological progression, and increases patient’s functioning [3,4]. These benefits are not achieved when patients are nonadherent to their long-term therapy [5,6]. It is estimated that around one-third of the patients with RA are nonadherent to antirheumatic drug therapy [7-9]. As such, achieving medication adherence remains a major challenge for a substantial proportion of patients with RA. Understanding medication nonadherence and its causes helps to identify targets for the development of adherence interventions. Practical barriers (eg, forgetfulness, costs) and patient’s attitudes toward medication (eg, balance between necessity and concerns) are associated with medication nonadherence [10,11]. Thus, these factors have frequently been the main target of interventions aiming to improve adherence [12]. Unfortunately, adherence interventions have been only partly effective [13-16].

Part of this ineffectiveness might be because the medication-taking behavior is not yet fully elucidated. Behavioral intentions such as taking medication are driven by a person’s explicit (conscious) and implicit (unconscious) attitudes [17]. These attitudes do not necessarily have to be congruent. Someone might explicitly say that medication helps alleviate symptoms but implicitly regard medication as chemical rubbish [17,18]. Habitual behavior, like medication-taking behavior, happens mainly on an unconscious level and is more likely to be guided by implicit attitudes [19]. Therefore, targeting implicit attitudes might be an effective strategy to improve medication adherence.

Implicit attitudes are targeted by reinterpretation training, that is, exercising the brain to interpret a stimulus differently [20]. This can, for instance, be achieved by performing tasks that lead to pairing of a medication stimulus with another positive stimulus [21]. Such a reinterpretation training needs rigorous and repetitive exercising to be successful or, in other words, a multidose intervention is required. eHealth can be a suitable mode of delivery for a multidose intervention as it is easily accessible and allows patients to perform these tasks at a convenient time and place. Retention of a multidose intervention is best achieved when participants are intrinsically motivated to prevent dropout prior to the effect of the intervention being reached.

Motivation can be maintained by formatting the intervention as a serious game [22,23]. Serious games are games that intend to entertain and achieve at least one additional goal [22]. In order to motivate patients to play the serious game, the self-determination theory may be used to guide serious game development. According to this theory, intrinsic motivation is most likely to occur when 3 needs are satisfied: competence, autonomy, and relatedness [24,25]. A serious game can satisfy these 3 needs, creating intrinsically motivated players who will adhere to a multidose intervention. Thus, serious games can positively influence behavior [26] even by targeting implicit attitudes [27].

Taken together, this paper describes the systematic development of a serious game by using the intervention mapping framework [28]. This serious game should provide entertainment as well as positively influence medication adherence by targeting implicit attitudes.

## Methods

### Development Process

Intervention mapping was used to systematically develop the intervention [29]. Intervention mapping considers and applies theory and empirical evidence to maximize the effectiveness and usability of the intervention, covers the complete range from problem identification to scientific evaluation, and ensures that the intervention is compatible with the target population [29]. A complex problem such as a medication-taking behavior demands a multidisciplinary approach. Therefore, the intervention mapping process was guided by meetings of an expert group consisting of a pharmacist, rheumatologist, rheumatology nurse, psychologist, innovation manager, representative of the pharmaceutical industry, and a game developer named Games for Health.

### Intervention Mapping Framework

The intervention mapping framework comprises 6 steps, where each step leads to a product that guides the subsequent step. See Table 1 for an overview of intervention mapping steps with associated tasks and intermediate development products. The goal of the first step is to assess the health problem. The main task in this step is to identify the determinants for the at-risk population of the problematic behavior (nonadherence). Step 2 builds on the previous step by using the identified determinants to formulate the change objectives. The change objectives specify who and what will change as a result of the intervention. In step 3, theory-informed methods and practical strategies are searched for that are most likely to accomplish the formulated change objectives. During step 4, the intervention is produced based on the outcomes of the previous steps and refined after pilot testing. The goal of step 5 is to increase program adoption, implementation, and maintenance by creating an implementation plan. Finally, in step 6, the effect of the intervention is evaluated to ensure that the desired behavioral outcome is achieved.

**Table 1:** Intervention mapping steps with associated tasks and applied methodology.

Intervention mapping steps	Intervention mapping tasks	Methods
Step 1: Logic model of the problem	Describe the context for the intervention Identify determinants for the at-risk population of the problem	PubMed literature search on determinants of nonadherence (2010-2015) Explorative study in 52 patients on relation between attitudes and medication adherence
Step 2: Program outcomes and objectives	State expected outcomes for behavior Specify performance objectives for behavioral outcomes Select determinants for behavioral outcomes Create a logic model of change	Multiple expert group discussions (both face-to-face and electronic)
Step 3: Program design	Generate program themes, components, scope, and sequence Choose theory- and evidence-based change methods Select or design practical apps to deliver change methods	Literature search and expert opinion on behavior change techniques Multiple expert group discussions Iterative game development
Step 4: Program production	Refine program structure and organization Prepare plans for program materials Draft messages, materials, and protocols Pretest, refine, and produce materials	Iterative game development Stage 1 user testing: 54 disease-modifying antirheumatic drug users played at home in 2 rounds for 2 weeks Stage 2 user testing: 8 disease-modifying antirheumatic drug users performed a live walk-through
Step 5: Program implementation plan	State outcomes and performance objectives for program use Construct matrices of change objectives for program use	Iterative game development guided by self-determination theory
Step 6: Evaluation plan	Write effect and process evaluation questions Develop indicators and measures for assessment Specify the evaluation design	Develop a randomized clinical trial study protocol to examine effectiveness on medication adherence of antirheumatic drugs (GAMER [Gaming for Adherence to Medication using E-health in Rheumatoid arthritis patients] study)

### Logic Model of the Problem

As the first step, the context of the intervention (population and setting) is described. Next, 2 methods were used to identify the determinants for patients with rheumatic disease being at-risk for nonadherence: (1) a literature search and (2) an explorative study on the implicit and explicit determinants toward antirheumatic drug use performed by research team members [30]. The literature search was performed in PubMed in 2015, and it focused on recent (2010-2015) studies, including systematic reviews, using the MeSH terms *medication adherence* and *rheumatic diseases* coupled with free text term *determinant*. Both primary studies and systematic reviews were included. All determinants mentioned in the selected studies and their association with medication adherence were collected and split into nonmodifiable and modifiable factors. Nonmodifiable factors aid in identifying the target population, whereas modifiable factors aid in identifying target behavior. Habitual behavior such as medication-taking behavior is likely to be guided by implicit attitudes as well as explicit attitudes [19]. However, it is unclear how explicit and implicit attitudes relate to medication adherence. Therefore, this was explored by research team members in a sample of patients with RA and published elsewhere [30]. In short, the sample consisted of 52 patients on oral methotrexate therapy at Sint Maartenskliniek, a Dutch tertiary rheumatology clinic. Patients were approached when collecting their medication refill, and assessment took place immediately after providing informed consent. Patients performed a computerized task (Single Category Implicit Association Test) to measure the implicit measures of medication attitudes and associations, which is a well-established and valid measure of implicit associations [31]. Additionally, they completed a questionnaire on demographics and questionnaires on explicit attitudes and associations (Beliefs about Medication Questionnaire [BMQ] [32-35]) and medication adherence (Compliance Questionnaire on Rheumatology [CQR] [36-38]), both proven valid and reliable in patients with RA. Clinical outcomes (erythrocyte sedimentation rate and C-reactive protein) were obtained from patients’ medical files. Because of the explorative character of this study, Pearson correlations were used to examine the relationship between patients’ explicit and implicit attitudes, associations, beliefs, adherence, clinical outcomes, and demographics.

### Program Outcomes and Objectives

The behavioral outcome of the intervention is to become adherent and maintain medication adherence of antirheumatic drugs. As the patient is the one who has the main influence on the medication-taking behavior, we only defined change objectives at the patient level. Thus, there are no change objectives at the interpersonal, organizational, communal, or societal level. The change objective of the intervention was guided by the outcomes of step 1 and established through multiple (electronic) discussions of the expert group through an organic iterative process.

### Program Design

The fundament of the behavioral change for our intervention was the Dual-Attitude model. The Dual-Attitude model postulates that implicit and explicit attitudes coexist and do not necessarily have to be congruent [17,30]. When dual attitudes exist, the implicit attitude is activated automatically, whereas the explicit one requires more capacity and motivation to retrieve from memory. As such, habitual behavior such as medication-taking behavior is more likely to be guided by implicit attitudes [19]. Implicit attitudes can be targeted by a behavior change technique called bias modification [20]. Google Scholar and PubMed were narratively searched for suitable behavior change techniques. The search terms consisted of free text words, that is, *behavior change technique*, *bias modification*, and *health*. To narrow the search results, the terms *review* and *overview* were added to the search strategy. The behavior change techniques shown to effectively address health behaviors were selected and presented to the game developer for applicability. Next, the game type was carefully chosen to suit the context (target population and setting) of the intervention from step 1.

### Program Production

The serious game was developed using an iterative design process. Based on the theory of the previous steps, the expert group prepared the outline of the intervention components in multiple sessions. Games for Health used their expertise to create the components within the technical possibilities and merged them to form the game. The game was tested by patients and the feedback used to adapt the game after which this process was repeated. Thus, the final product is a practical interpretation of the theory. The test panel members were representative of the target group and were recruited from Sint Maartenskliniek, Nijmegen, The Netherlands. They were patients aged 16 years or older who used antirheumatic drugs. Ethical approval for user testing was asked for and waived by the local medical research ethics committee of Arnhem-Nijmegen under code 2017-3355. A random sample of 500 patients using DMARDs received an invitation with informed consent enclosed through mail. Additionally, participants needed to possess a tablet and be proficient in the Dutch language.

Stage 1 consisted of 2 rounds of 2 weeks of user testing at home after which data on acceptability were collected. Acceptability was determined using the Technology Acceptance Model as underpinning, which is a well-established model for usability evaluation of eHealth [39-41]. This model postulates that ease of using a technology influences the perceived usefulness and the attitude toward using and together form the behavioral intention to use a technology, which leads to actual use. Ease of use was measured using the System Usability Scale questionnaire taken directly from the Technology Acceptance Model [39,42,43]. The perceived usefulness of a game was operationalized as enjoyment and assessed using the GameFlow questionnaire, which has been successfully applied to distinguish between the high-rated and low-rated games and identify why one succeeded and the other failed [44,45]. Attitude toward using was assessed using 4 questions of the user version of the Mobile App Rating Scale (uMARS), which is a simpler end-user version of the validated MARS [46,47]. The questions of the uMARS that captured the overall feeling of the app and its potential use were selected by authors BPHP and BJFvdB until consensus was reached. All other questions were omitted, as they related to other aspects of mobile apps and even overlapped with ease of use and usefulness. Actual use was collected using Google Analytics and determined to be time played and number of sessions. In addition, participants were asked for their overall experience and suggestions for improvement (open-ended questions) to inform the game developers.

Stage 2 was a live walk-through where patients performed tasks within the serious game environment under supervision. A team of game developers from Games for Health and author BPHP observed the participants and took notes. Participants were recruited from players in stage 1 (experienced users) and from the patient representatives of Sint Maartenskliniek (new users). Suggestions for improvement were collected with the aim of improving gameplay and increasing retention.

### Program Implementation Plan

Intrinsic motivation is key to ensure adoption and implementation of a serious game. The self-determination theory posits that motivation is a continuum between extrinsic motivation (ie, external factors such as rewards or grades) and intrinsic motivation (ie, internal factors such as interest, curiosity, or care). Intrinsic motivation can be reliably enhanced by supporting the satisfaction of 3 psychological needs: competence, autonomy, and relatedness [24,25,48]. Competence denotes the experience of mastery. It becomes satisfied when capably engaging in activities and experiencing opportunities for using and extending skills. Autonomy denotes the experience of willpower and willingness without external pressure. Relatedness denotes the experience of bonding and care and is satisfied by connecting to others. In the Results section, we have described how our serious game addresses these needs.

### Evaluation Plan

To assess whether the developed intervention positively affects antirheumatic drug adherence, a research proposal was drafted for a multicenter randomized controlled trial: the GAMER (Gaming for Adherence to Medication using E-health in Rheumatoid arthritis patients) study.

## Results

### Logic Model of the Problem

The intervention is set within the context of RA. RA mainly affects people older than 50 years and is more common among women [1]. Because most antirheumatic drugs are used at home, our adherence-enhancing intervention should be utilized in the home setting. The literature search on determinants of nonadherence resulted in 73 publications, of which 12 detailed on determinants of medication adherence in rheumatic diseases [7,10,11,49-57]. There were no nonmodifiable patient characteristics that indisputably predicted medication nonadherence. Therefore, we decided that our intervention should be aimed at all patients with RA. The modifiable determinants that remained were psychosocial and therapy-related factors. As our intervention should not interfere with RA treatment, we focused on psychosocial factors. Supportive evidence was found for the following modifiable psychosocial factors influencing medication adherence: perceived treatment necessity, treatment concerns, satisfaction with care, treatment self-efficacy, coping, practical barriers, social support, disease or treatment understanding, illness beliefs/perceptions, and lifestyle. The necessity/concerns balance and practical barriers had the strongest association with medication adherence [10,53]. As stated in the introduction, behavioral intentions are driven by both explicit (conscious) and implicit (unconscious) attitudes [17]. Habitual behavior such as medication taking is guided stronger by implicit attitudes than by explicit attitudes, which play a stronger role in conscious (planned) behavior [19]. To understand the possible role of implicit attitudes regarding medication-taking behavior, we performed an explorative study with 52 patients who showed that explicit attitudes were positive and health-related. Implicit attitudes were, however, negative and illness-related. Half of the patients displayed explicitly positive but implicitly negative attitudes [30]. The relationship between implicit attitudes and medication adherence is worth being further explored to potentially make interventions more effective.

### Program Outcomes and Objectives

The primary outcome of the intervention is to become adherent and maintain adherence to antirheumatic drugs, which was defined as taking at least 80% of the prescribed doses. This cutoff is widely used in (RA) adherence research and associated with improved in clinical outcomes in RA [5]. It is increasingly recognized that medication adherence is not an order from a clinician for the patient to execute (“compliance” to therapy) but requires active patient participation and stimulation (adherence). Thus, an intervention enhanced with positive affect is more successful in increasing adherence [58]. In addition, the explorative study learned that patients’ implicit and explicit attitudes do not correlate and that implicit attitudes are generally negative and illness-related. Therefore, the expert group considered that reconditioning implicit negative attitudes to more positive ones could shift the necessity/concerns balance. In that light, the expert group drafted a change objective that was adjusted and refined over several rounds of discussion. Ultimately, this led to the following change objective: after the intervention, participants have a more positive attitude toward antirheumatic drugs.

### Program Design

The explorative study in patients with RA performed in step 1 learned that, generally, explicit attitudes are positive and implicit attitudes are negative [30]. To enable change to occur, the expert group aimed at reducing negative explicit attitudes and reinforcing positive implicit attitudes (see Table 1). The idea was that the net result of these 2 actions would be overall a more positive attitude toward medication. Medication concerns can be targeted by patient education [12,51]. Thus, our strategy was to explicitly reduce concerns by educating patients on how to best use antirheumatic drugs. The literature search on bias modifications to change implicit attitudes led to multiple reviews with examples of gamified behavior change techniques [20,21]. To positively influence the associations between medication beliefs and medication use on an implicit level, 3 mental domains can be addressed: cognition (knowing), affect (feeling), and motivation (willing) [20].

*Cognitions/beliefs* can be altered using attentional bias modification training [21]. During training, attention is shifted in a positive direction by repetitively drawing attention to positive associations between medication beliefs and medication use. Similarly, *affect* can be modified by training participants to pair medication with another positive stimulus—so called evaluative conditioning. Lastly, *motivation* can be implicitly targeted by goal priming: passive and unobtrusive activation of people without them being aware of it. Taken together, we applied 1 explicit and 3 implicit strategies as underpinning for behavior change to occur. Implicit attitudes are activated automatically, but like old habits, are harder to change [17]. Thus, a multidose intervention in the form of a serious game was chosen. The expert group identified game types that fit the target population, which in the case of RA are mainly women over the age of 50 years. One of the favorite leisure time activities is solving puzzles, and therefore, it was decided to develop a serious puzzle game [59,60].

### Program Production

**Figure 1:**
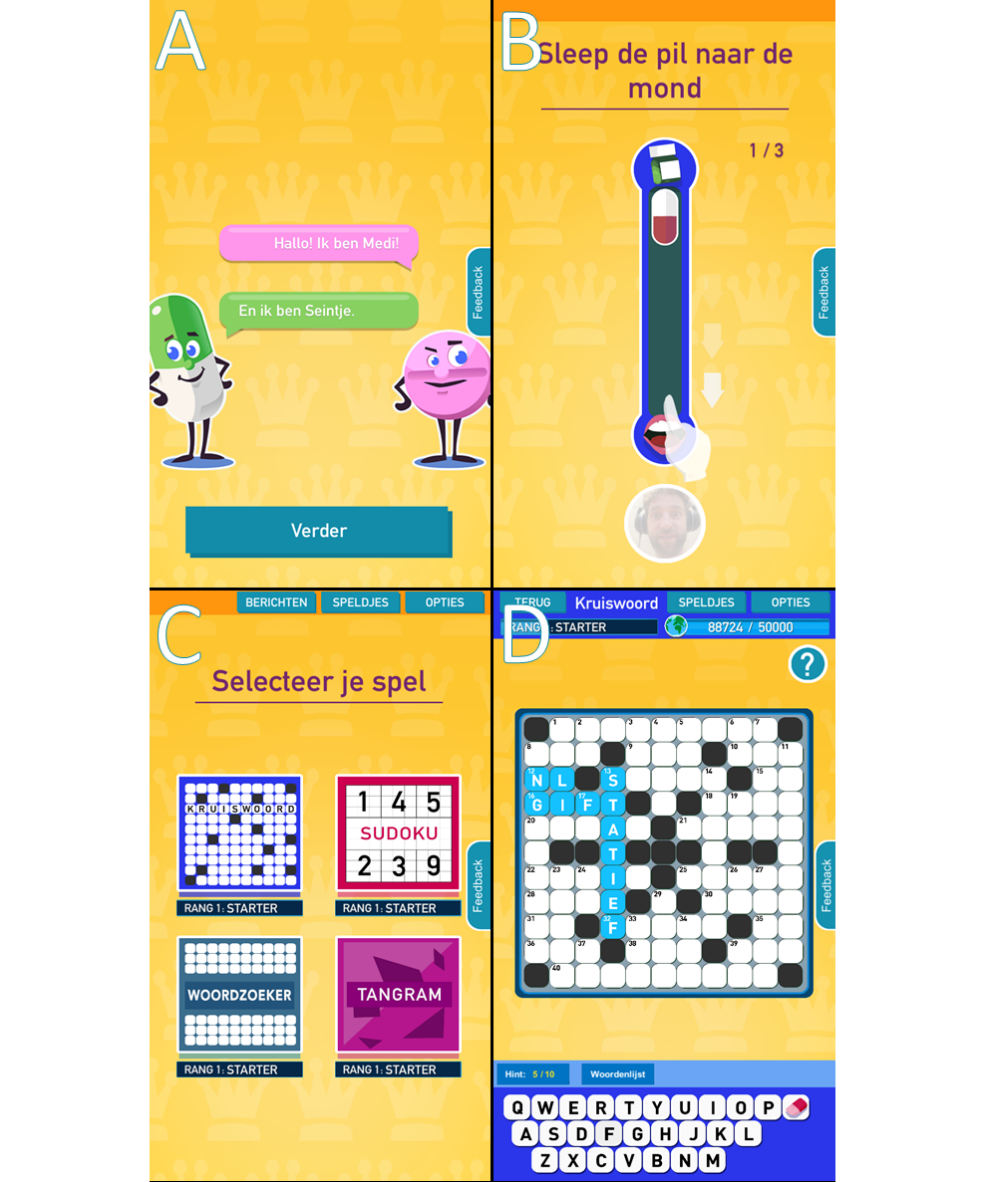
Screenshots of the serious puzzle games. A. Icon characters Medi and Seintje introduce themselves. B. Users are instructed to slide the pill down the screen toward a picture of the user to unlock trigger. C. The puzzle menu showing the 4 puzzle types: crossword, sudoku, word search, and tangram. D. Example of the crossword puzzle screen.

The design of the game environment needed to merge medication and puzzles and simultaneously be positive and energizing. The game was named Medi and Seintje, which is a Dutch wordplay on medication and signaling. Medi and Seintje are icon characters that look like a tablet and capsule, respectively (see Figure 1A). To ensure that participants would relate to the game, game personification was built in. If participants allowed camera use, they could take a picture of themselves and of their medication, which was used in the behavior change techniques (see below). Next, the behavior change techniques had to be integrated into the puzzle game in such a way that participants would encounter them without being too obtrusive to disturb gameplay. The behavior change techniques were added to the puzzle environment as so called “triggers” that allowed participants to open the game or a puzzle. A total of 5 triggers were developed: multiple choice medication quiz, dot-probe task, visual search, slide to unlock (see Figure 1B), and a barcode scanner (see Multimedia Appendix 1). These triggers were gamified behavior change techniques and considered important game components (see Table 1). After completing the trigger at start-up, the game offered 4 puzzle types (see Figure 1C and Figure 1D), each with 3 levels of difficulty: crossword, sudoku, wordsearch, and tangram. The game environment adhered to the Medi and Seintje theme. The first 4 steps of intervention mapping have been summarized in Table 2 and Table 3.

**Table 2:** From change objective to intervention strategies—the first 2 steps of intervention mapping.

Step	Goal	Outcome
Step 1	Determinants	Treatment necessity Treatment concerns
Step 2	Change objective	After the intervention, participants have a more positive attitude toward antirheumatic drugs.

**Table 3:** From change objective to intervention strategies—step 3 and step 4 of intervention mapping.

Step, goal		Strategies and outcomes			
		1	2	3	4
**Step 3**					
	Domain	Motivation-implicit	Affective-implicit	Cognitive-implicit	Cognitive-explicit
	Strategy	Goal priming: passive, subtle, and unobtrusive activation by external stimuli such that people are not aware of the influence exerted by those stimuli.	Reinforcing the positive valence of antirheumatic drug use by strengthening the positive associations through pairing antirheumatic drugs with a positive stimulus.	Reinforcing attention toward medication using positive stimuli. Part of the techniques applied are based on attention bias modification training.	Reduce concerns by educating patients on how to best use antirheumatic drugs.
**Step 4**					
	Game component	Personalization of the game Icon characters “Medi and Seintje” Come-and-play reminder Barcode scanner	Personalization of the game Icon characters “Medi and Seintje” Energizing/positive gaming environment Slide to unlock Visual search	Personalization of the game Icon characters “Medi and Seintje” Dot-probe task Visual search	Multiple choice medication quiz

Out of 500 invitations, 54 DMARD users (11%) agreed to test the game at stage 1. Their median age was 63 years and the median number of years since diagnosis was 10 years. Thirty-three participants were female (61%) and 39 (72%) used their tablet daily. Stage 1 consisted of 2 rounds, where the feedback of round 1 was incorporated in the game before testing in round 2. Of the 52 participants, 39 participants completed the study: 9 participants did not download the app (reason unknown), 2 stopped owing to technical issues, and 2 stopped because of medical reasons. In round 1, 19 participants used the app and 22 participants used the app in round 2, of which 12 used the app in both rounds. On average, in round 1, users played 1.4 sessions per day that lasted 12 minutes, and in round 2, users played 1.7 sessions per day that lasted 16 minutes. Although playtime increased, there were no significant differences in the scores for ease of use, perceived usefulness, and attitude toward using between the 2 rounds. User experiences indicated a broad spectrum of views from joy from playing to annoyance. Suggestions for improvements given by participants were mainly about the barcode scanner, as the scanner malfunctioned in round 1. Other technical improvements that were suggested were a lower frequency of push notifications, larger display buttons, and preventing puzzles from causing the app to crash. Prior to the live walk-through in stage 2, the app received a major update to incorporate further improvements, such as instruction screens for all puzzles. During stage 2, eight DMARD users performed a walk-through under supervision at Sint Maartenskliniek. Four participants participated in stage 1, and 4 were new to the app. When seeing how users performed the various tasks, the app builders learned which steps were intuitive and which steps needed improvement. Overall, the design process led to valuable insights in patient acceptance, usability, and suggestions for improvement. Consequently, the latest version of the app complied with the needs of end users.

### Program Implementation Plan

Implementation was ensured by evoking the intrinsic motivation of participants through addressing the following 3 needs: competence, autonomy, and relatedness [24,25,48]. The complete puzzle environment consisted of 3 puzzle types—crossword, sudoku, and wordsearch—with 3 levels of difficulty and at least 50 puzzles at each of these levels and 82 tangram puzzles across 4 themes: animals, letters, objects, and humans. To meet the need for *competence*, puzzles with increasing difficulty were available. Players could board a puzzle on the difficulty level they could master and develop skills by playing numerous puzzles in increasing difficulty. For players new to the game, there was an option to receive hints or help. The mastery of an individual was tracked by gaining experience points when playing puzzles, and they could view their progression level. Additionally, players could complete challenges such as “find a word within 5 seconds” after starting wordsearch to be rewarded with badges allowing them to track and visualize their progress.

To meet the needs of *autonomy*, players had the freedom to choose which puzzle to play (individual choices were reflected in the badges collected) and the opportunity to solve a puzzle in multiple ways. Finally, to meet the need of *relatedness,* the world record playing crossword puzzles was incorporated in the game. By playing crossword puzzles, each player contributed to breaking the world record crossword puzzles, which was a group effort. Prior to starting a new crossword puzzle, the individual’s contribution to the world record and total progress was shown. To protect the privacy of the individual participants, it was decided not to incorporate social interaction elements at this stage. To further prevent dropout, we sought to balance triggers versus puzzles. Balance turned out to be one trigger when starting the game and when opening a new puzzle after at least 10 minutes of solving puzzles. Triggers appeared in random order to maintain variety in gameplay.

### Evaluation Plan

The intervention is currently being evaluated in a multicenter randomized clinical trial: the GAMER study [61]. This study aims to examine the effect on medication adherence and clinical outcomes in patients with RA treated with antirheumatic drugs. A total of 220 patients will be randomized 1:1 to intervention or usual care and followed for 3 months. The intervention group will be instructed to install and play the puzzle game on their tablet or mobile phone. Playing the puzzle game is encouraged at the start of the study but otherwise completely voluntary. The main study parameter is adherence using the validated CQR in an intention-to-treat analysis. Additionally, a pill count will be performed and the BMQ will be collected. Secondary clinical outcomes are the Health Assessment Questionnaire (HAQ) and the self-reported Rheumatoid Arthritis Disease Activity Index (RADAI). The CQR, BMQ, HAQ, and RADAI have been proven valid and reliable in patients with RA [32-38,62-66]. Disease activity [67,68] will be gathered if available. Lastly, the Technology Acceptance Model, a well-established model for evaluating usability of eHealth, will be applied to collect patient acceptance of the puzzle game. Data collection will be similar to stage 1 of the user testing: the System Usability Scale will assess ease of use, GameFlow will assess perceived usefulness, part of the uMARS will assess the attitude toward using, and Google Analytics will collect actual use [39-46].

## Discussion

This paper describes the design rationale of a serious game aimed at improving medication adherence in patients with RA. Our formative work with patients with RA in combination with the literature search and explorative study described above led us to develop a mobile serious game as an intervention. Focal points of this serious game were implicit medication attitudes, positivism, and retention.

As Abraham et al [69] stated, development of serious games should detail on the extent of the theoretical framework incorporated into the game design and evaluate success by testing the player’s retention of learning objectives. This is why we chose to develop our intervention according to the intervention mapping framework while being guided by the Dual-Attitude Model and self-determination theory [17,24]. Even though the development was guided by the systematic intervention mapping framework, several choices still had to be made by the expert group. To ensure deliberate decisions, we sought to incorporate many different areas of expertise among group members from clinical to psychological and technical. Patients were not represented in the expert group but extensively consulted throughout the intervention mapping process: from the explorative study to elaborate user testing. The developed intervention did not contain medication-taking (reminder) components in contrast to other serious games aimed at improving medication adherence [69]. We decided not to incorporate the actual medication-taking behavior because we feared that this would be perceived as coercive and would lead to loss of retention because the act of medication taking would take playfulness and positivity out of the game.

The behavior change techniques we have applied as medication-related triggers have not previously been tested to improve medication adherence. Even though there is no solid evidence for improving medication adherence, the extensive research on these techniques for stimulating healthy behavior was considered a strong enough premise to apply these techniques in our serious gaming intervention [21]. Another reason for applying these behavior change techniques was the fact that they have been successfully and effectively gamified [26,27]. It should be noted that the test conditions for these behavior change techniques were generally well-controlled: playing the gamified behavior change techniques for a set period of time (at least for several minutes) without distractions. When applying these techniques in a mobile app as medication-related triggers, there is no control over the participants’ setting, which leads to variable exposure to the triggers. To ensure that the triggers were sufficiently dosed, participants need to be intrinsically motivated to play the game. When developing a serious game, a trade-off has to be made between the serious (ie, the behavior change techniques) and the game (ie, the puzzles), which is why the usability testing is so important. The results from our usability testing indicated a positive response toward the app. However, these findings were prone to selection bias and limited to patients willing to test the app. This type of testing, while appropriate for app development, may not reveal barriers to implementation in practice. The app was carefully designed to quickly engage users, sustain motivation for long-term app use, and simultaneously apply behavior change techniques. The success of these strategies will not be known until the app is tested in clinical practice. To be considered effective, serious games must sustain their impact over the long term and offer more than a short-term novelty effect [69]. The results of our evaluation study will hopefully answer if our serious game is successful in improving medication adherence [61]. If proven effective, additional studies should be performed to assess effectiveness in the longer term (6-12 months) and to investigate the effective components more closely.

In conclusion, we systematically developed a serious game app to enhance adherence to antirheumatic drugs among patients with RA by using the intervention mapping framework. Evaluation in a multicenter randomized controlled trial will determine intervention uptake and effectiveness. This paper could serve as a guideline for other health care providers when developing similar interventions.

## Appendix

Multimedia Appendix 1 Triggers of serious game Medi and Seintje explained.

## Abbreviations

BMQ: Beliefs about Medication Questionnaire
CQR: Compliance Questionnaire on Rheumatology
DMARD: disease-modifying antirheumatic drugs
GAMER: Gaming for Adherence to Medication using E-health in Rheumatoid arthritis patients
HAQ: Health Assessment Questionnaire
RA: rheumatoid arthritis
RADAI: Rheumatoid Arthritis Disease Activity Index
uMARS: user version of the Mobile App Rating Scale
