# Supplementary material for: A Serious Puzzle Game to Enhance Adherence to Antirheumatic Drugs in Patients With Rheumatoid Arthritis: Systematic Development Using Intervention Mapping
Source: JMIR Serious Games. 2022 Feb 18;10(1):e31570. doi: 10.2196/31570 (PMC8900908; doi:10.2196/31570)
Supplement: Multimedia Appendix 1 [file games_v10i1e31570_app1.docx]

Triggers of serious puzzle game ‘Medi & Seintje’

*This document describes the five triggers employed by serious puzzle game ‘Medi en Seintje’. These triggers are gamified behaviour change techniques. The serious game aimed to influence medication taking behaviour.*

##
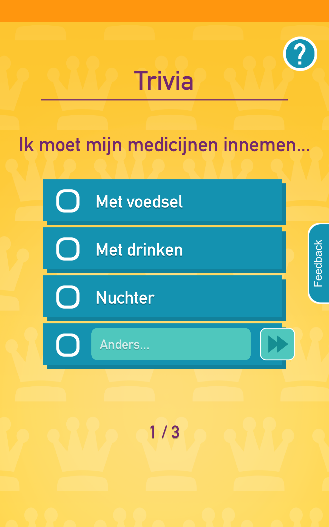
Multiple choice medication quiz

**Domain** Explicit cognition.

**Strategy** Reduce concerns by educating patients on how to best use antirheumatic drugs.

**Based on** education as means to improve medication adherence

**Frequency** Three questions per event

**Description** A set of multiple choice questions was developed as an entertaining cognitive task to shift the necessity/ concern balance by educating on practical medicine information like how to best swallow a pill.

##
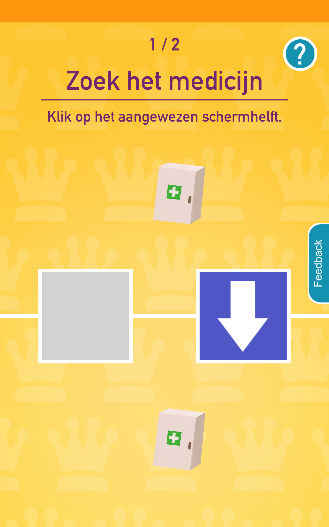
Dot-probe task

**Domain** Implicit cognition.

**Strategy** Adjust the automatic beliefs by strengthening positive associations. Part of the techniques applied are based on attention bias modification training.

**Based on** dot-probe

**Frequency** Two runs per event

**Description** On the screen icon character ‘Medi’ is shown standing between two empty squares. The instruction is to focus on icon character ‘Medi’ while a countdown from 3 to 1 is shown in the squares. After the countdown a stimulus is shown for 500 milliseconds in both squares after which the positive stimulus is replaced by an arrow pointing up or down while simultaneously a medication cabinet appears on the upper and lower part of the screen. The player is instructed to select the medication cabinet to which the arrow is pointing to find the medication. When the correct cabinet is selected, the medication jumps out.

**Options** Medication cabinet is either a regular cabinet or a refrigerator. Players are able to use a picture of their own medication.

## **
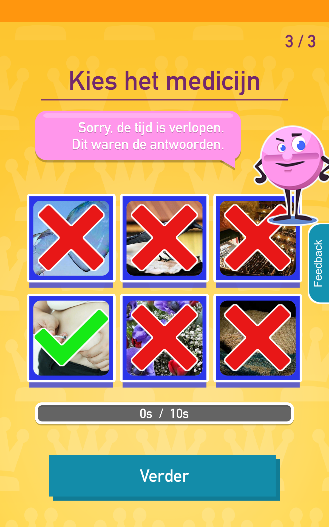
**Visual search

**Domain** Implicit cognition and affect.

**Strategy** Adjust the automatic beliefs by strengthening positive associations. Part of the techniques applied are based on attention bias modification training. Adjust the valence of anti-rheumatic drug use by strengthening the positive associations through pairing anti-rheumatic drugs with a positive stimulus.

**Based on** visual search task: attention is drawn to medication (cognition) in an array of other positive stimuli (affect).

**Frequency** Three runs per event.

**Description** (Similar to reCAPTCHA) Players were tasked with finding medication in an array of six or nine pictures (all positive) within 10 seconds. If no or wrong input is given, the correct answers are shown after 10 seconds. The picture database consists of positive images (eg, the sun, smiling children) and rheumatological medication (eg, methotrexate blister, someone injecting a biological). The picture set could be supplemented with a picture of the player’s medication.

**Options** Six or nine pictures shown. Single or multiple medication images. Players are able to use a picture of their own medication.

## **
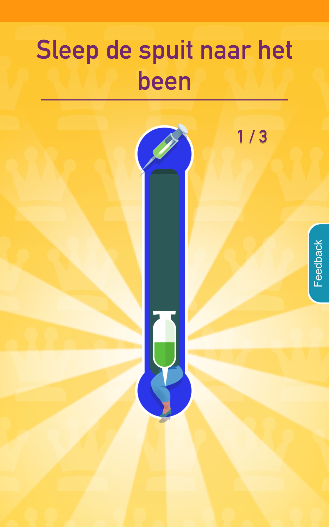
**Slide to unlock

**Domain** Implicit affect.

**Strategy** Adjust the valence of anti-rheumatic drug use by strengthening the positive associations through pairing anti-rheumatic drugs with a positive stimulus.

**Based on** approach-avoidance task aimed at inducing approach or avoidance behaviour by simulating attraction and repulsion respectively

**Frequency** Three runs per event.

**Description** Players were instructed to swipe medication towards the bottom of the screen. The approach effect was stimulated by increasing the size along the way. At the bottom of the screen a picture starts to become clearer as the medication is drawn near. When the bottom is reached, the task is accomplished which was visualised by showering the medication in rays.

**Options** Either pill/mouth or syringe/leg combination. Players are able to use a picture of themselves.

##
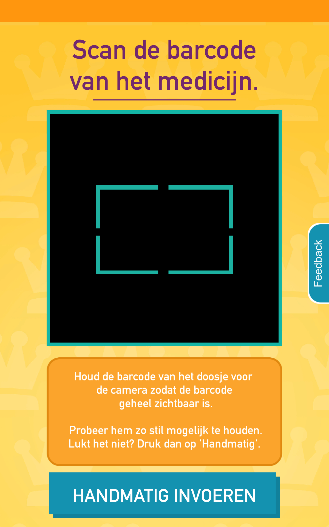
Barcode scanner

**Domain** Implicit motivation.

**Strategy** Goal priming: passive, subtle, and unobtrusive activation by external stimuli such that people are not aware of the influence exerted by those stimuli.

**Based on** -

**Frequency** Once per event.

**Description** The barcode scanner literally motivated players to engage with the medication because the app would only unlock if a barcode of the medication was scanned. This feature was only active if the game had access to the camera. If medication was not at hand or failed to scan, players could manually enter the barcode.
